# Roles of *CcDFR* and *CcOMT9* in the cyanidin biosynthesis and development of *Cordyceps cicadae*

**DOI:** 10.3389/fmicb.2024.1353710

**Published:** 2024-03-06

**Authors:** Zixuan Zeng, Yu Zou, Weiming Cai, Fu-Cheng Lin, Hongkai Wang

**Affiliations:** ^1^State Key Laboratory of Rice Biology, Institute of Biotechnology, Zhejiang University, Hangzhou, China; ^2^Institute of Horticulture, Zhejiang Academy of Agricultural Sciences, Hangzhou, China; ^3^State Key Laboratory for Managing Biotic and Chemical Treats to the Quality and Safety of Agro-products, Institute of Plant Protection and Microbiology, Zhejiang Academy of Agricultural Sciences, Hangzhou, China

**Keywords:** *Cordyceps cicadae*, cyanidin-3-O-glucoside, growth and development, bioactive substance, gene function

## Abstract

**Introduction:**

*Cordyceps cicadae* is a traditional Chinese medicinal fungus known for its rich production of bioactive substances, particularly cyanidin, an anthocyanin commonly found in plants with notable anti-inflammatory, anti-tumor, antiviral, and antibacterial properties. This study revealed two key genes, *CcDFR* and *CcOMT9*, affecting cyanidin biosynthesis in *C. cicadae*.

**Methods:**

The roles of these genes in cyanidin production, growth, and development were elucidated through the gene knockout method, phenotypic analysis, transcriptomics, and metabolomics.

**Results:**

*CcDFR* deletion led to reduced cyanidin-3-O-glucoside (C3G), suppressed expression of cyanidin biosynthesis genes, impaired synnemata formation, decreased polysaccharide and adenosine content, and diminished chitinase activity. Meanwhile, the *ΔCcOMT9* mutant exhibited an increase in C3G production, promoted expression of cyanidin biosynthesis genes and rising bioactive compounds, suppressed RNA methylation, and led to phenylalanine accumulation with no effect on fruiting body formation.

**Discussion:**

We revealed a distinct anthocyanin biosynthesis pathway in *C. cicadae* and identified two genes with opposite functions, laying the foundation for future genetic modification of cyanidin-producing strains using modern biological techniques. This will shorten the production period of this valuable compound, facilitating the industrial-scale production of cyanidin.

## Introduction

*Cordyceps cicadae*, a parasitic fungus that hosts cicada nymphs, is widely distributed throughout China and other Asian countries and possesses significant medicinal and nutritional value, resulting in its popularity in traditional Chinese medicine and supplements. Numerous studies have demonstrated its ability to enhance immune regulation activity, reduce inflammation, and inhibit tumor growth (Layland et al., 2014; Wei et al., 2016; Meng et al., 2019; Nxumalo et al., 2020). In addition, several studies have demonstrated the potential of *C. cicadae* as a microbial insecticide against various insects, such as aphids (Xu et al., 2020). Its natural composition is akin to that of *Cordyceps sinensis*, with active components primarily comprising polysaccharides, nucleosides, sterols, fatty acids and their derivatives, polyphenols, etc. (Hsu et al., 2002; He et al., 2018; Meng et al., 2019). To date, new bioactive compounds are continually discovered from this useful fungus (He et al., 2018; Xie et al., 2020). But there are few reports involving natural pigments other than carotenoids.

Cyanidin, the most widely distributed source of anthocyanins in plants, is a flavonoid compound that serves as a natural pigment. It possesses various biological activities and medicinal value, including anti-inflammatory, anti-tumor, and anti-oxidation effects (Chen et al., 2005; Xia et al., 2005; Ding et al., 2006; Duchnowicz et al., 2012; Serra et al., 2013; Wang et al., 2017a; Li et al., 2020). The biosynthesis of cyanidin has been investigated in plants, encompassing both the phenylpropanoid metabolic pathway and flavonoid anabolic pathway (Chen et al., 2016; Wang et al., 2017b; Li et al., 2018). The synthesis of anthocyanidins undergoes three stages from phenylalanine: first, L-phenylalanin ammonialyase (PAL) catalyzes the conversion of phenylalanine to cinnamic acid and then the formation of 4-coumaryl-CoA is regulated by cinnamic acid-4-hydroxylase (C4H) and 4-coumarate-CoA ligase (4CL) (Boss et al., 1996). The second stage is the biosynthesis of colorless anthocyanin from 4-coumaryl-CoA, involving chalcone synthase (CHS), chalcone isomerase (CHI), flavonone-3-hydroxylase (F3H), flavonoid-3′-hydroxylase (F3’H), flavonone-3′-5′-hydroxylase (F3’5’H), and dihydroflavonol-4-reductase (DFR) (Forkmann and Dangelmayr, 1980; Froemel et al., 1985). The third stage involves the synthesis of cyanidin, whereby colorless anthocyanidin is transformed into colored cyanidin through anthocyanidin synthase (ANS); subsequently, stable C3G is formed following a series of glycosylation, methylation, and acylation modifications (Holton and Cornish, 1995; Gonzalez et al., 2008). The biosynthesis pathway of cyanidin in fungi, however, has received little attention. Bu et al. reported a novel discovery of an anthocyanin synthesis pathway in *Aspergillus sydowii* H-1. Together, 31 genes were identified through transcriptomics and metabolomics for directing cyanidin production (Bu et al., 2020). DFR is a crucial component in the biosynthesis of anthocyanins, belonging to the NADPH-dependent short-chain reductase family or DFR subfamily that encodes single genes. The members of this subfamily are represented by cinnamic acid and oxidoreductase (Martens et al., 2002). DFR is a key enzyme in the plant anthocyanin biosynthetic pathway that plays an important role in flower color development, as the differences in DFR expression and its substrate specificity determine the color variation in flowers (Zhao and Tao, 2015). Although the DFR homologs are found in the genome of many fungi, their functions are rarely uncovered.

Methyltransferases (MTs) are a class of pivotal enzymes that are widely distributed in animals, plants, and microorganisms, playing an indispensable role in the regulation of growth, development, and secondary metabolism (Bohnsack et al., 2019; Li et al., 2022a; Ramdhan and Li, 2022). In RNA methylation modifications, SAM is commonly utilized as a methyl donor for methylation reactions, thereby regulating cellular growth and development, repairing gene damage, and facilitating the synthesis and degradation of metabolites (Liscombe et al., 2012). RNA modifications are dynamic and reversible processes, comprising m5Cs and m6As (Bohnsack et al., 2019; Oerum et al., 2021). RNA methyltransferases (MTases) are the “writer” enzymes to m6A/m5C. There are seven MTase members in humans which are responsible for m5Cs in RNAs, while there are only three members in yeast. However, for m6As, there are multiple RNA MTases in mammals, but it is generally believed that there is only one in Ascomycetes (Xu et al., 2017). The m6A is found to participate in post-transcriptional modification of mRNA in yeast, plants, flies, humans, and other mammals and bacteria (Oerum et al., 2021). In addition to mRNA, m6A is also found in ribosomal RNA (rRNA) from both mammals and bacteria but not in yeast or archaea. Structures of RNA MTases are available for functional characterization from humans, zebrafish, and bacteria (Oerum et al., 2021). To date, little is known about the relationship between RNA methylation modification and anthocyanidin synthase. The gene function of RNA methylation related to growth and compound metabolism is unclear in *C. cicadae*.

The fungus *C. cicadae* can develop a fruiting body known as coremium/synnemata which is the main part used as food and medicine for people (Li et al., 2022b), but the molecular genetic mechanisms of synnemata formation are rarely studied. Song et al. utilized phenotypic and transcriptomic analysis to elucidate the role of the blue light receptor gene, *Icwc1*, in affecting hyphal growth and fruiting body (synnemata) development in *C. cicadae* (Song et al., 2023). Li et al. have demonstrated how the GPI-anchored protein homolog, IcFBR1, regulates synnemata development, secondary metabolism, and nutrient utilization (Li et al., 2022c), thus providing valuable insights into the genetic regulatory mechanism’s control over fungal development. Investigation into the growth and development of *C. cicadae* is instrumental in uncovering its biological characteristics and adaptation strategies, thus providing a scientific foundation for its cultivation and rational utilization.

It is of significant scientific importance and potential application to further explore the active substances of *C. cicadae* and study the molecular mechanisms underlying its biosynthesis. During the research on molecular mechanisms of metabolism and development in *C. cicadae*, we discovered two genes regulating the ability of cyanidin biosynthesis. In this study, two genes relating to cyanidin biosynthesis in *C. cicadae* were investigated in detail. Through phenotypic and omics analyses, we studied the synthesis, growth and development, active components, and bacteriostatic ability of cyanidin. Furthermore, we predicted the molecular mechanism underlying its biosynthesis. These findings provide a theoretical basis and technical means for resource development and utilization of *C. cicadae*.

## Materials and methods

### Strains and culture conditions

*Cordyceps cicadae* strain 2-2, stored at Zhejiang University, was cultured in YES medium (Supplementary Table S8) for 10 days at 25°C. The vector pKO1B for knockout and pKD5 for complementation was stored in our laboratory. *Escherichia coli* DH5α (Sangon Biotech, Shanghai) for plasmid preservation and *Agrobacterium tumefaciens* AGL1 (Tsingke Biotechnology, Beijing) for *Agrobacterium*-mediated transformation (ATMT) were cultured in LB medium at 37°C and 28°C, respectively. The preserved strains and bacteria were stored at −80°C with the addition of 20% glycerol.

### The knockout and complementation of *CcDFR* and *CcOMT9*

Using the principle of homologous recombination, we utilized the pKO1B vector, including both upstream and downstream homology arms, to the target gene as well as *HPH* fragments for knockout vector construction. To get a complementation vector, we also constructed the pKD5 vector including the target gene. U1/U2 and D1/D2 primers were used to amplify the 1.0–1.5 kb upstream and downstream fragments of the target gene from the WT genome. Validation primers KOCX/MRBF and Trptrcx/cmcx2 were used to verify the vectors, respectively. The transformation of bacteria was achieved by the freeze-thaw method. The fungal transformation procedure was conducted in accordance with the methodology described by Li et al. (2022a). The knockout mutants were confirmed as a single copy via qPCR using the 2^–∆∆CT^ method and were repeated three times. The primers used in the above experiments are listed in Supplementary Table S1.

### The establishment of an extraction-assay system for C3G from *Cordyceps Cicadae*

Because cyanidin is not stable for detection and transfers to cyanidin-3-O-glucoside (C3G) quickly in cells, we detected the amount of C3G, which is a stable and downstream substance of cyanidin (Bu et al., 2020).

**The extraction of C3G**: the mycelium was introduced into the CM liquid medium (Supplementary Table S9) for incubation in a shaker (25°C, 150 rpm) over a period of 2 days. A measurement of 1.0 g of freeze-dried mycelia samples was collected and mixed with 10 mL of a 1% hydrochloric acid-methanol solution. The samples were subjected to 24 h ultrasonic cleaning for fixing extraction. The resulting filtrate was obtained by filtering through three layers of filter paper and transferring 1.0 mL into a 25 mL brown volumetric bottle. Prior to sample testing, the filtrate was shaken with methanol and filtered through a 0.25 μm microporous filter membrane.

**The determination of C3G**: a series of cyanidin-3-o-glucoside control solutions were prepared at concentrations ranging from 0.5 to 25.0 μg/mL and filtered through a 0.25 μm microporous filter membrane before testing. Phenomenex Synergi Hydro-RP column (250 mm × 4.60 mm, 5 μm) was used. The mobile phase consisted of 0.5% phosphoric acid solution as phase A and water acetonitrile as phase B (50:50, V/V). Gradient elution was carried out with the following program: 0 min (18%B), 23 min (50%B), 28 min (18%B), 33 min (18%B), and 40 min (stop); flow rate: 0.8 mL/min; detection wavelength: 520 nm; column temperature: 30°C. The standard solution of each volume concentration was repeated three times to obtain the corresponding peak area (Supplementary Figure S1B). A standard curve was constructed using C3G concentration as the abscissa and its corresponding peak area as the ordinate, with a linear regression equation calculated. The resulting regression equation between C3G concentration and peak area was *y* = 20.559*x* − 2.3947 (*R*^2^ = 0.9998), demonstrating a strong linear correlation within the range of 0.5–25 μg/mL (Supplementary Figure S1C).

### Functional analysis of *CcDFR* and *CcOMT9* in *Cordyceps Cicadae*

**The content of the active ingredient**: *polysaccharide*: the standard curve was drawn using the glucose standard, the polysaccharide was extracted by the phenol sulfuric acid method, and the OD value was determined at 490 nm. *Adenosine*: the standard curve was generated through HPLC analysis of adenosine standards. Phenomenex Synergi Hydro-RP column (250 mm × 4.60 mm, 5 μm) was used. The mobile phase consisted of 0.5% phosphoric acid solution as phase A and methanol as phase B. Gradient elution: 0 min (100%A), 13 min (85%A), 30 min (40%A), and 40 min (stop); flow rate: 1 mL/min; detection wavelength: 260 nm; sample size: 20 μL; column temperature: 25°C. The standard curve of adenosine was obtained by plotting the volume concentration of adenosine on the horizontal axis and its corresponding peak area on the vertical axis, resulting in a linear equation *y* = 122.8*x* − 7.0017 (*R*^2^ = 0.9999). The adenosine was extracted by sonication and methanol. *Chitinase and Anthocyanin*: Chitinase and anthocyanin were analyzed using the kit (Boxbio, Beijing) and microplate reader.

**The rate of growth**: the 5 mm agar disks with mycelium were inoculated on a 7 cm YES plate medium and incubated at 28°C under alternating conditions of 16 h of light and 8 h of darkness. After 8 days, the colony diameter was measured.

**The yield of spores**: the 5 μL of spore suspension was inoculated on a 9 cm plate PDA medium and cultured for 9 days. A measurement of 4 mL of ddH_2_O was added to the plate, and the spores were washed with a spreader before being filtered through three layers of filter paper to obtain the spore solution. The yield of spores was determined by observing and counting with a hemocytometer and microscope.

**The mycelial growth under chemical stress**: the 5 mm agar disks with mycelium were inoculated on the YES media supplemented with different chemicals, including 100 μM menadione (VK_3_), 1 mM hydrogen peroxide (H_2_O_2_), rose Bengal (RB), 0.6 g/mL Congo red (CR), 0.4 M sodium chloride (NaCl), 0.4 M potassium chloride (KCl), and 0.5 M Sorbitol. The colony diameters were also measured at 8 days post incubation at 25°C in the dark. Each group was repeated three times with five replicates.

**The formation of fruiting body**: the strains were inoculated onto a sterilized medium of wheat seeds. The fruiting body formation was observed after dark culture for 7 days and light culture for 2 weeks at 25°C.

**The bacteriostatic ability**: *Pseudomonas solanacearum*, *Xanthomonas oryzae*, *Dickeya dadantii* NCPPB 898, and *Escherichia coli* were inoculated on NA solid medium. The crude extracts of wild type and mutant, each with a volume of 50 μL, were added into the 5 mm hole of the medium. The diameter of the inhibition zone was recorded after incubation at 28°C overnight.

### The effects of *CcDFR* or *CcOMT9* deletion on the expression of genes involved in cyanidin synthesis pathway in *Cordyceps Cicadae*

The genes related to the synthesis pathway of cyanidin were identified by the local blast (Supplementary Table S1), and the expression levels were analyzed by RT-qPCR.

### Metabolomics analysis of the effects of *CcDFR* and *CcOMT9* on the synthesis pathway of cyanidin in *Cordyceps Cicadae*

In this study, the non-targeted metabolomics analysis of secondary metabolites was conducted on WT and mutants *ΔCcDFR* and *ΔCcOMT9* using liquid chromatography-mass spectrometry (LC–MS) technology (Bioincloud, Shenzhen), and the specific analytical method was done according to the methodology described by Shan et al. (2023).

### Transcriptome analysis of the effects of *CcOMT9* on the synthesis pathway of cyanidin in *Cordyceps Cicadae*

Mycelium was harvested from YES plate medium after being cultured for 10 days at 25°C (16 h light and 8 h darkness) and total RNA was extracted. The cDNA and RNA libraries were sequenced according to previous research (Chen et al., 2018). Ribosomal reads were removed by bowtie2, and the remaining total reads were used for subsequent transcriptome analysis. Total Reads (-rRNA) were aligned with the genome of *C. cicadae* using HISAT2 software. DESeq2 (Love et al., 2014) software was utilized for differential expression analysis between the control and treatment groups. Differentially expressed genes (DEGs) were identified based on FDR < 0.05 and |log2FC| > log2(2), with up-regulated and down-regulated DEGs being obtained. MeRIP (Methylated RNA Immunoprecipitation), based on the principle of m6A-specific antibody recognition and binding to m6A-modified mRNA, is a technique used for enriching methylated RNA followed by high-throughput sequencing analysis to study regions of m6A methylation modification across the entire transcriptome. MACS2 (version: 2.1.2) software was used to identify read-enriched regions from MeRIP-seq data (Zhang et al., 2008). Dynamic Poisson distribution was used to calculate the *p*-value of the specific region based on the unique mapped reads. The region would be defined as a peak when the *q*-value <0.05. The DiffBind (version 2.8) software was used to analyze the RNA methylation rate between groups (Stark and Brown, 2011). The relative methylation rate of each peak was calculated using MeRIP data and input data. We identified peaks with log2|FC| ≥ 1 and FDR ≤ 0.05 in a comparison as significant differential peaks for subsequent peak-related genes GO and KEGG enrichment analysis. GO and KEGG databases were employed to significantly enrich all DEGs and differential peaks in the WT and mutant *ΔCcOMT9*, thereby identifying major metabolic and signal transduction pathways.

## Results

### The validation of knockout and complementation mutants

The *C. cicadae* strain 2-2 can secrete red pigment to medium when inoculated on PDA. We found that C3G, the important anthocyanin, could be detected by HPLC (Supplementary Figure S1A). In the process of investigation on the functional genes related to growth, development, and metabolism, we found two genes in strain 2-2 genome, marked as FUN_1459 and FUN_9127 in our Lab, involved in red color pigment biosynthesis, for the mutants of the two genes, changed color when inoculated in PDB after gene knock-out. Gene FUN_1459 contains 1,142 bp, including 1 intron, and encodes 353 amino acids (accession No. C_AA042572.1). The protein sequences with high similarity and the sequences of model organisms were collected from NCBI and analyzed by the neighbor-joining method (NJ) to establish the phylogenetic tree (Supplementary Figure S2). FUN_1459 has the highest homology to *Cordyceps fumosorosea* ARSEF 2679 dihydroflavonol-4-reductase (accession No. XP_018706651.1), followed by dihydroflavonal-4-reductase in *Cordyceps javanic* (accession No. TQW08783.1), *Beauveria brongniartii* RCEF 3172 (accession No. OAA49002.1), and *Cordyceps militaris* CM01 (accession No. XP 006670355.1). However, it is hard to say whether FUN_1459 is homologous to plants like *Arabidopsis thaliana*, *Pyrus communis*, and *Dianthus caryophyllus*. When blast analysis was performed in the genome of *Aspergillus sydowii* H-1, we revealed that FUN_1459 gene was homologous with DFR_1; the key gene encoded dihydroflavonol-4-reductase in the anthocyanin biosynthesis pathway (Bu et al., 2020), and we named FUN_1459 as *CcDFR* (accession No. C_AA042572.1). Gene FUN_9127 contains 1,521 bp, including 1 intron, and encodes 351 aa. Blastp showed that the homologous protein of FUN_9127 is all putative methyltransferase and actin-binding protein in fungi. As shown in the phylogenetic tree, FUN_9127 has the highest homology to methyltransferase (Trm140) in *Beauveria bassiana* (accession No. PMB67945.1), followed by acting-binding protein in *Trichoderma simmonsii* (accession No. QYT05915.1). It seems there was no obvious homology to *Saccharomyces cerevisiae* S288C (accession no. Q08641.3) and *Schizosaccharomyces pombe* 972 h- (accession no. Q9P7L6.2). There is no study on *B. bassiana* about Trm140. However, in consideration of Trm140 as a methyltransferase responsible for 3-methylcytidine modification in the tRNA anti-codon loop in model organism *S. cerevisiae* (accession no. Q08641.3), we named FUN_9127 as *CcOMT9* (accession no. C_AA042573.1).

**Figure 1:**
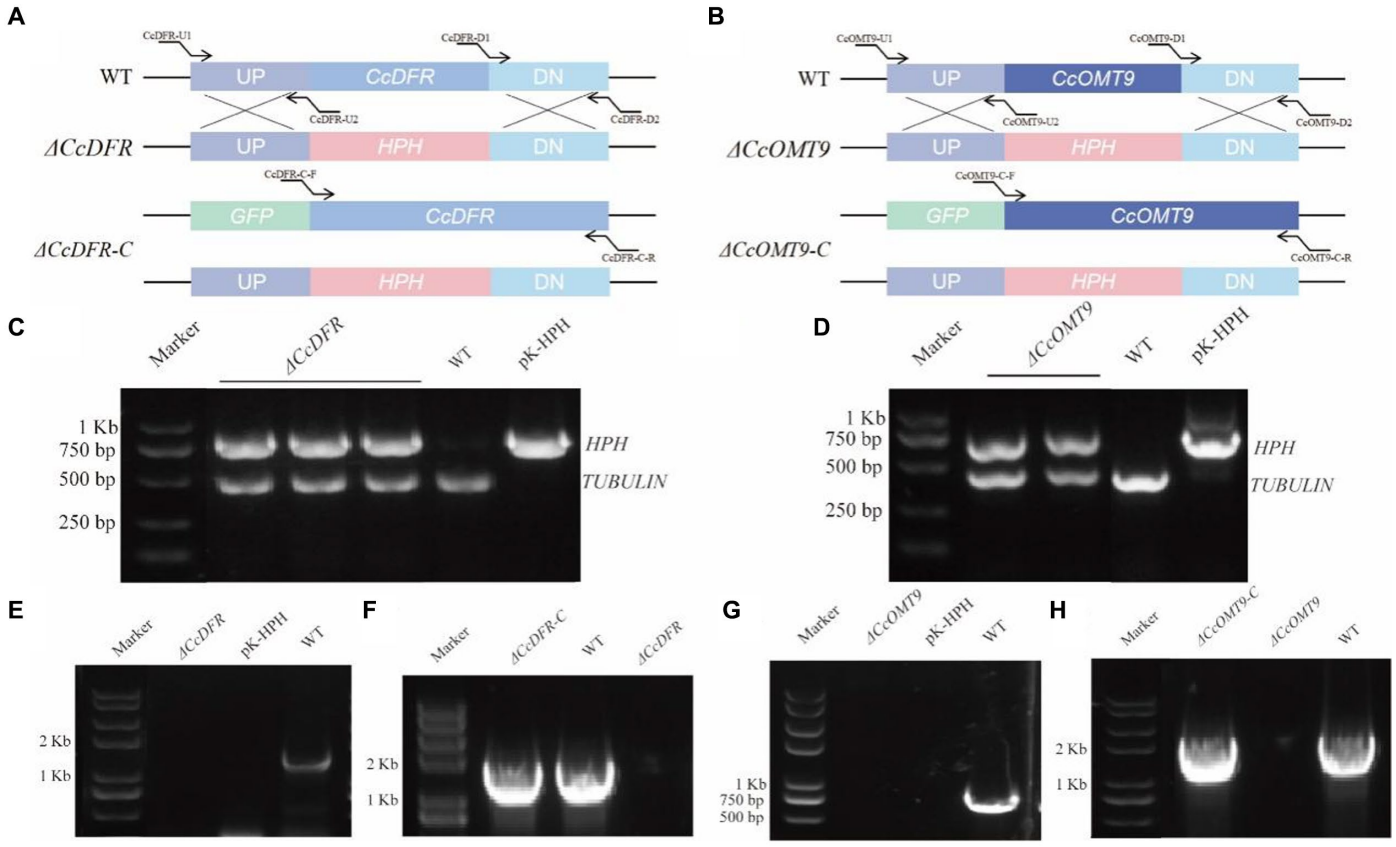
The schematic diagram of gene knockout and complementation of target genes in *C. cicadae* **(A,B)**. The schematic diagram of *CcDFR* and *CcOMT9* knockout and complement construction **(C,D)**. The first validation of knock-out vector **(E,G)**. The second validation of the knockout mutants and the complemented strains **(F,H)**. The validation of *ΔCcDFR*-C and *ΔCcOMT9*-C mutants.

Knockout vectors and their complementary vectors with corresponding resistance were obtained based on the schematic diagram in Figure 1 and transformed by the ATMT method. The mutants exhibiting hygromycin B resistance generated with ATMT were validated following PCR (Figures 1A,B). The single copy of the mutant strains was determined by RT-qPCR. The complementation mutant was confirmed by obtaining a band size identical to that of the WT (Figures 1C–H).

### *CcDFR* and *CcOMT9* exert differential effects on the production of C3G

**Figure 2:**
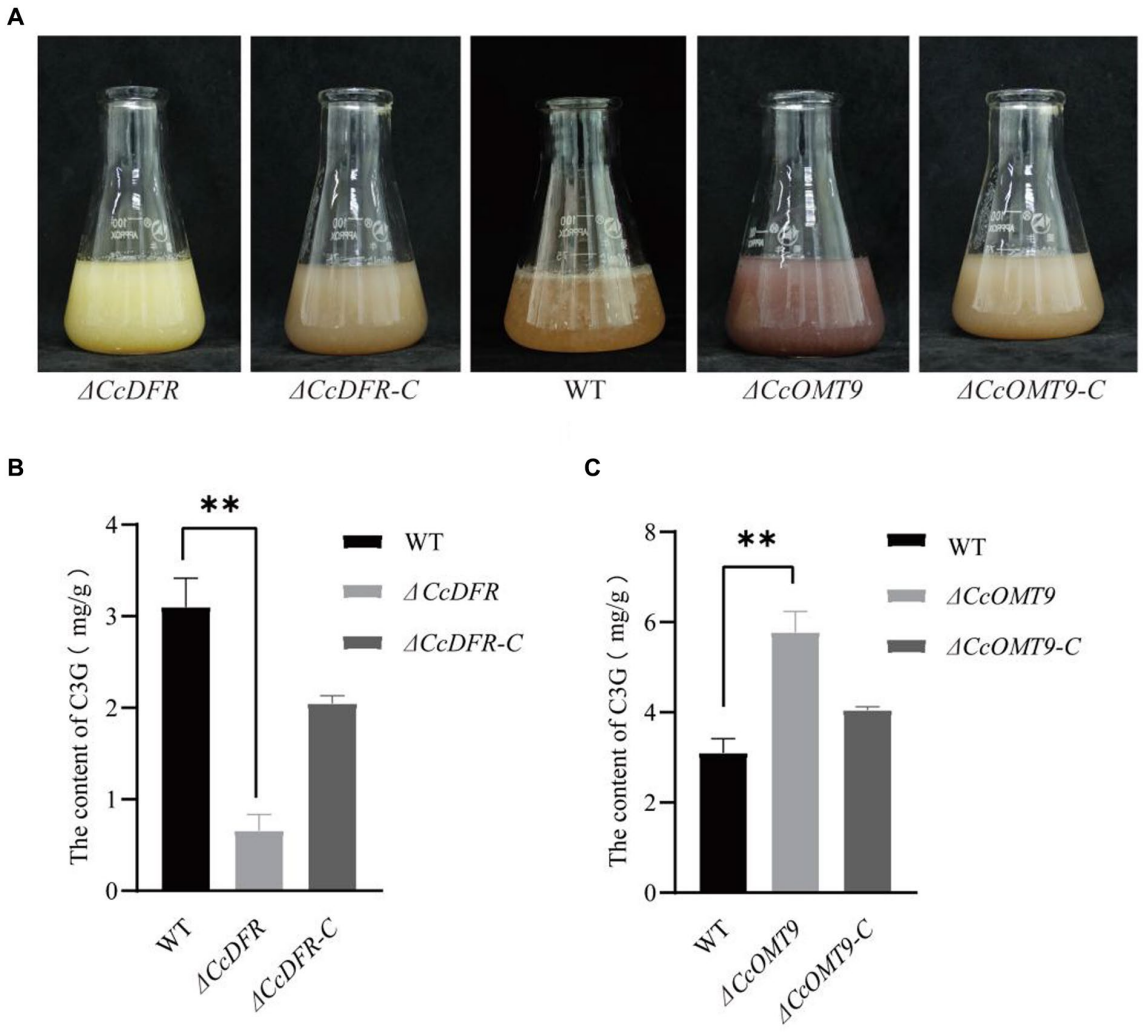
The quantitative analysis of C3G content. **(A)** The growth status of WT and mutants in the liquid PDB medium for 4 days. **(B,C)** The content of C3G in WT and mutants. The experiment was then repeated three times independently, taking the final average and performing one-way ANOVA using SPSS and LSD Duncan after fact comparison. *p ≤ 0.05 and **p ≤ 0.01 indicate statistically significant data; NS indicates no significant difference.

During the process of liquid fermentation, notable distinctions in color were observed between the WT and *ΔCcDFR* mutants (Figure 2A). Upon knockout of methyltransferase-related genes, we observed significant differences between the *ΔCcOMT9* mutant and WT, with the former having a more obvious red pigment production (Figure 2A).

To further substantiate that *CcDFR* and *CcOMT9* are influencing C3G yield and resulting in color change, we conducted quantitative analysis via HPLC (Shirley et al., 1995). The C3G content of the *ΔCcDFR* mutant was 0.7194 ± 0.17 mg/g and the *ΔCcOMT9* mutant was 5.7748 ± 0.50 mg/g, while the WT was 3.1016 ± 0.31 mg/g (Figures 2B,C).

### *CcDFR* and *CcOMT9* affect the antibacterial ability

Previous research demonstrated the antibacterial ability of *C. cicadae*, cyanidin, and C3G (Gopu and Shetty, 2016; Zhang et al., 2017; Cen et al., 2021; Li et al., 2022b). The inhibitory zone method was employed to assess the antibacterial ability, and the findings indicated that the inhibitory zone of *ΔCcDFR* mutant was significantly reduced compared to that of WT. *C. cicadae* exhibits inhibitory effects against *P. solanacearum*, *X. oryzae*, *D. dadantii* NCPPB 898, and *E. coli*, and knockout of *CcDFR* significantly reduces the inhibitory zone size. Specifically, the inhibitory zone against *P. solanacearum* was 27.23 ± 0.03 mm, while that of WT was 31.33 ± 0.05 mm. The inhibitory zone of *ΔCcDFR* was 28.28 ± 0.05 mm for *X. oryzae* and 30.53 ± 0.06 mm for WT, indicating that *CcDFR* plays a crucial role in regulating the antibacterial activity of *C. cicadae* (Supplementary Table S2).

*ΔCcOMT9* exhibited a significantly larger inhibitory zone compared to WT treatment. Knockout of *CcOMT9* significantly increased the inhibitory zone size against *P. solanacearum*, *X. oryzae*, *D. dadantii* NCPPB 898, and *E. coli*, indicating that *CcOMT9* affects the antibacterial activity of *C. cicadae*, while knockout of *CcDFR* significantly decreased the inhibitory zone size (Supplementary Table S2).

### *CcDFR* and *CcOMT9* affect the production of bioactive components

**Figure 3:**
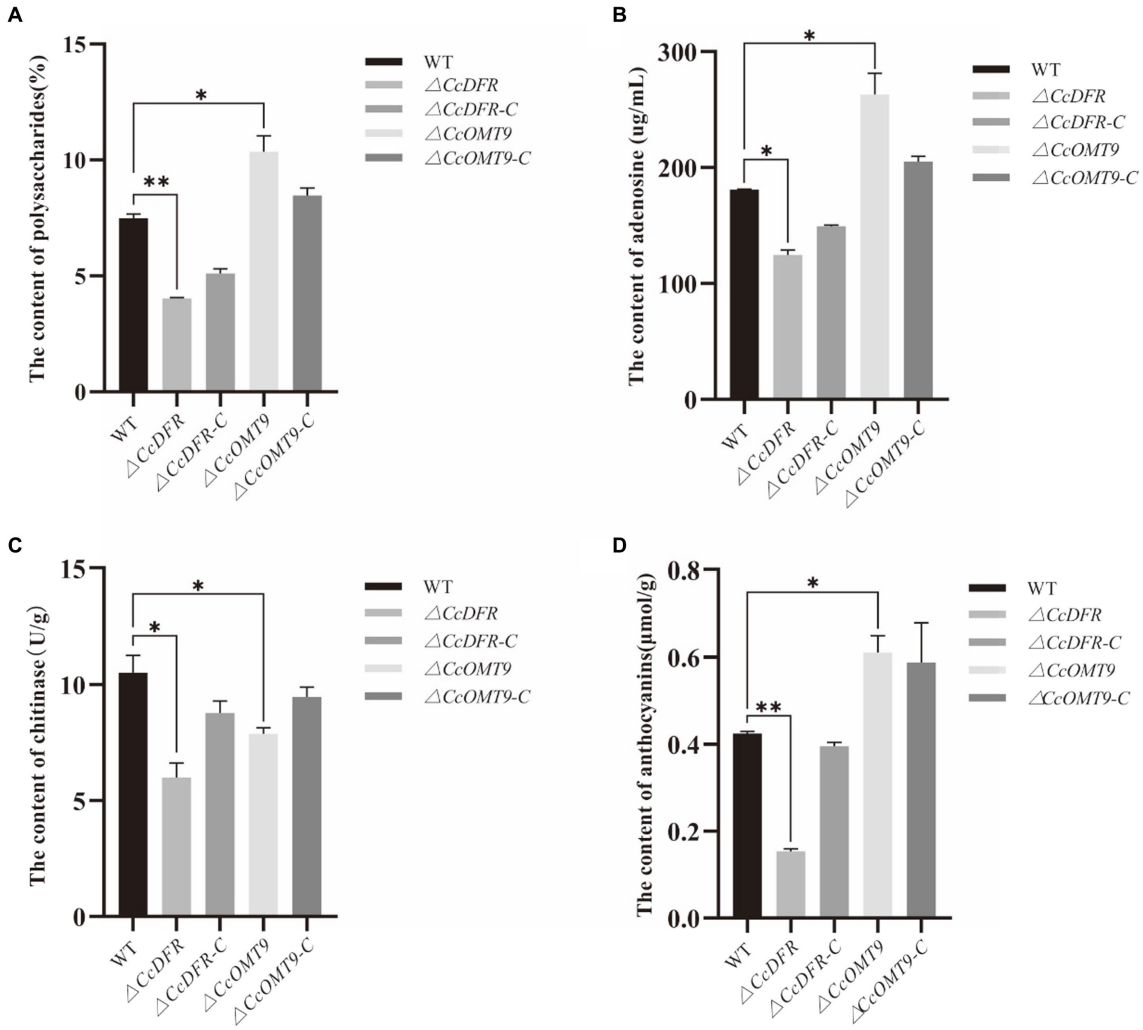
The effect of *CcDFR* and *CcOMT9* on the bioactive components of *C. cicadae*. **(A)** The effect of *CcDFR* and *CcOMT9* on polysaccharide production; **(B)** The effect of *CcDFR* and *CcOMT9* on adenosine content; **(C)** The effect of *CcDFR* and *CcOMT9* on chitinase activity; **(D)** The effect of *CcDFR* and *CcOMT9* on anthocyanin content.

Compared to the WT, the *ΔCcDFR* and *ΔCcOMT9* mutant showed significant differences in polysaccharide content, while the former was down-regulated measuring 4.02 ± 0.04% and the latter was up-regulated measuring 10.36 ± 0.67%, which was a decrease of 46.33% and an increase of 38.32% from the WT’s value of 7.49 ± 0.17%, respectively (Figure 3A). The effect of *CcDFR* on adenosine production was analyzed by HPLC. Following ultrasonic extraction of adenosine, deletion of *CcDFR* resulted in a significant reduction in adenosine content for *ΔCcDFR* mutant compared to WT, with the former yielding 124.51 ± 4.44 μg/mL and the latter yielding 180.86 ± 0.52 μg/mL, representing a decrease of 31.16%. Deletion of *CcOMT9* resulted in a significant increase in adenosine content for *ΔCcOMT9* mutant compared to WT, with the former yielding 263.2 ± 18.09 μg/mL and the latter yielding 180.86 ± 0.52 μg/mL, representing an increase of 45.65% (Figure 3B). Compared to the WT chitinase activity of 10.52 ± 0.75 U/g, the chitinase activity of *ΔCcDFR* mutant was significantly reduced by 43.06% to only 5.99 ± 0.63 U/g, and *ΔCcOMT9* mutant was significantly reduced by 25.10% to only 7.88 ± 0.26 U/g (Figure 3C). The content of anthocyanins in the *ΔCcDFR* mutant was significantly reduced by the pH-differential method (Figure 3D). The *ΔCcDFR* mutant exhibited a significantly lower content of anthocyanins at 0.15 ± 0.0056 μmol/g, representing a reduction of 65.12% compared to 0.43 ± 0.0043 μmol/g of WT, while the *ΔCcOMT9* mutant exhibited a significantly higher content of anthocyanins at 0.61 ± 0.04 μmol/g, representing an increase of 41.86% compared to 0.43 ± 0.0043 μmol/g of WT.

### *CcDFR* and *CcOMT9* exert an influence on the growth, development, and fruiting body formation

**Figure 4:**
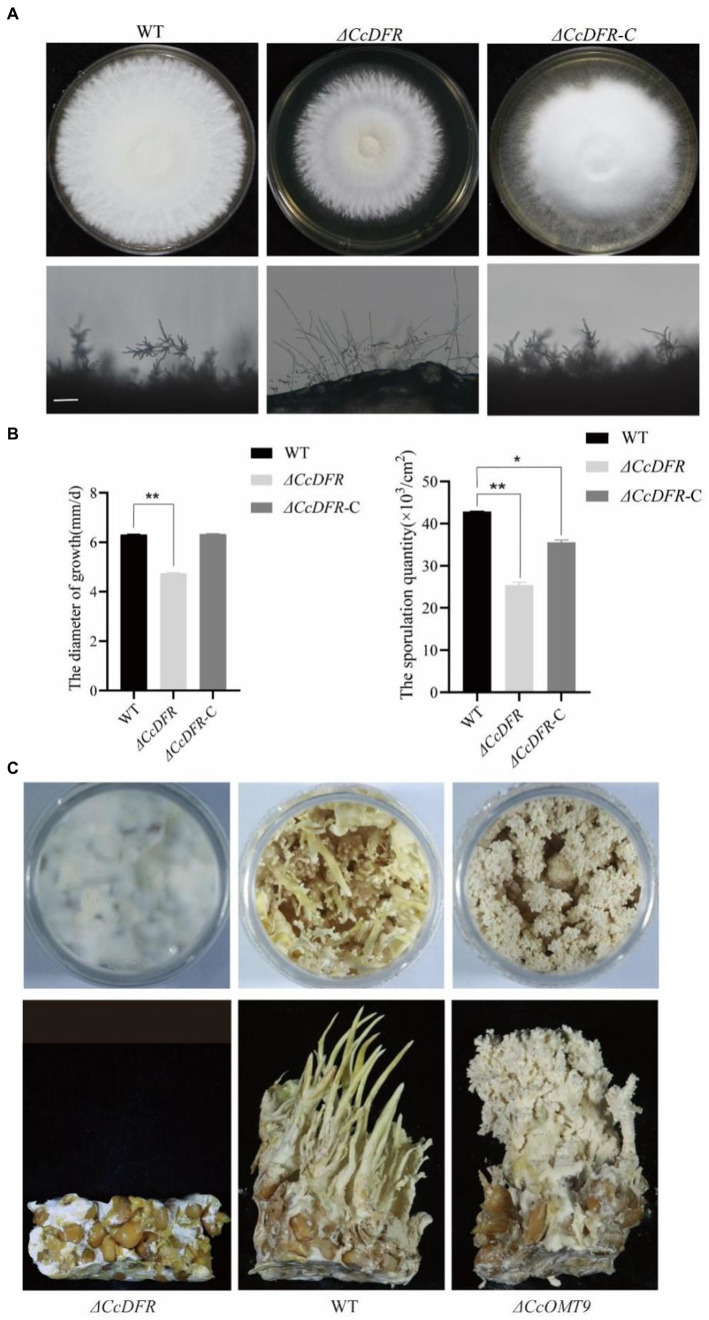
The effect of the *CcDFR* and *CcOMT9* on the growth and development of *C. cicadae*. **(A)** The colony morphology and differentiation status of WT and mutants after 10 days on YES medium. Bar = 50 μm; **(B)** The sporulation of strains after 10 days of culture on YES medium; **(C)** The effect of *CcDFR* and *CcOMT9* on the formation of fruiting bodies in *C. cicadae*.

Compared to the WT and complementation strain, the *ΔCcDFR* mutant exhibited a significant reduction in colony diameter after 10 days of growth on YES medium at 25°C, with a decrease in growth rate of 25.4% compared to the WT strain. Additionally, thinner aerial mycelium was observed and the sporulation yield decreased by 78.26% relative to the WT (Figures 4A,B).

During the fruiting body stage, it is observed that the *ΔCcOMT9* mutant exhibits precociousness, shorter synnemata length, lighter color, and earlier “flowering” compared to the WT, whereas the *ΔCcDFR* mutant fails to produce fruiting bodies (Figure 4C), indicating that *CcDFR* plays a crucial role in regulating spore production and growth rate as well as promoting fruiting body formation during *C. cicadae* development.

### *CcDFR* and *CcOMT9* affect the resistance to cell wall stress

**Figure 5:**
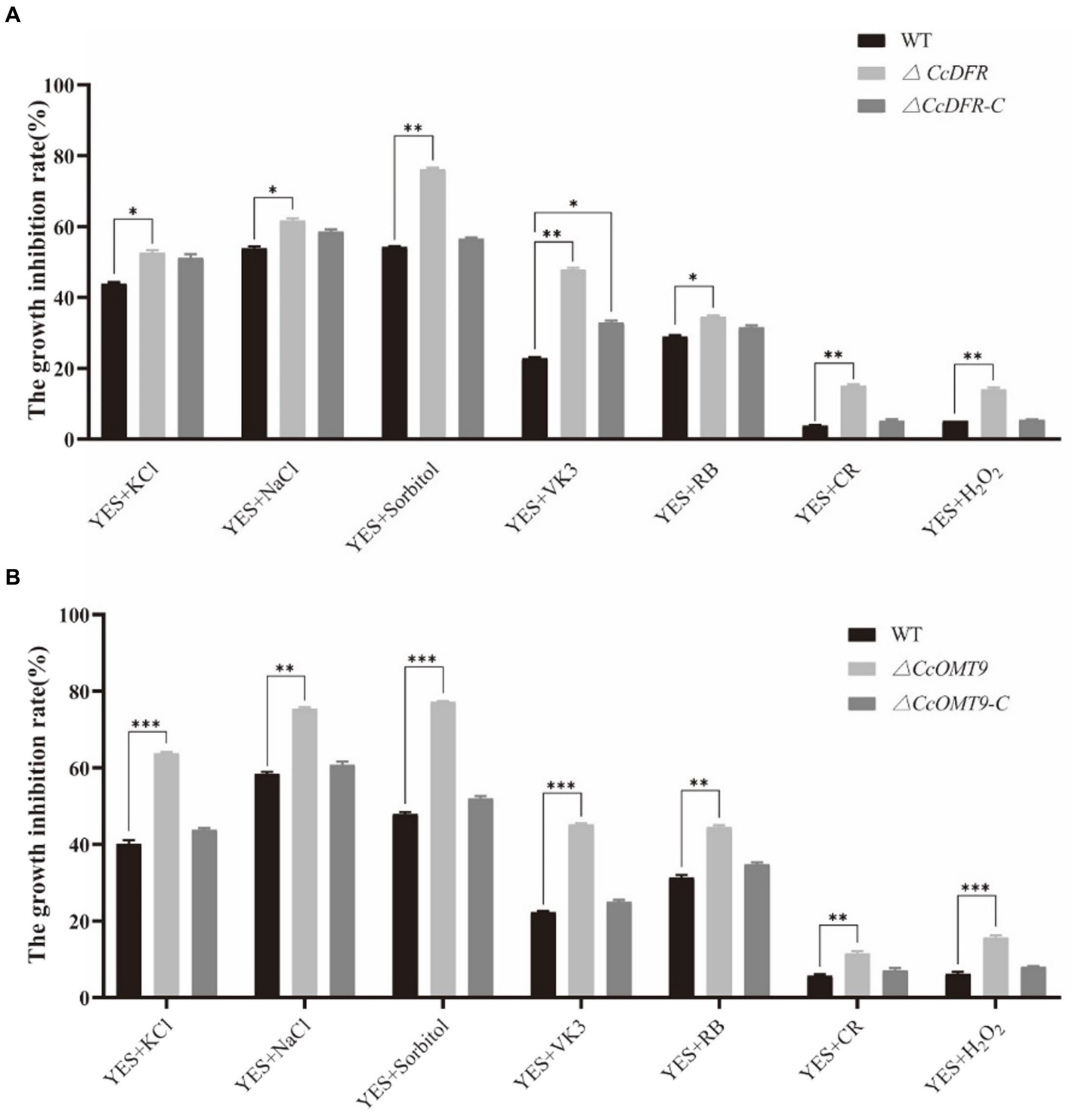
The effect of *CcDFR* and *CcOMT9* on stress resistance of *C. cicadae*. **(A,B)** The relative inhibition rate of WT and mutants under different stress factors (the relative inhibition rate = the diameter on the YES plate/the diameter on the YES plate). The experiment was then repeated three times independently, taking the final average and performing one-way ANOVA using SPSS and LSD Duncan after fact comparison. *p ≤ 0.05 and **p ≤ 0.01 indicate statistically significant data; NS indicates no significant difference.

In the process of growth, differentiation, reproduction, and invasion, *C. cicadae* will be stressed by the external environment. Compared with the WT (54.27 ± 2.5%), Sorbitol has more significant inhibition on the *ΔCcDFR* mutant (76.74 ± 2.2%). On the YES + RB condition, the growth inhibition rate of the mutant (48.17 ± 1.7%) was observed to be significantly higher than that of the WT (34.05 ± 4.1%) (Figure 5A). The inhibitory rate of KCl for WT was 40.13 ± 0.92% while the *ΔCcOMT9* mutant was 68.30 ± 0.30%. Moreover, on the YES + VK3 plate, the growth inhibition rate of the *ΔCcOMT9* mutant (45.21 ± 0.26%) was also markedly greater than that of the WT (22.29 ± 0.34%) (Figure 5B). These results indicated that *CcDFR* and *CcOMT9* play an important role in the resistance to cell wall stress.

### *CcDFR* and *CcOMT9* affect the expression of genes related to the cyanidin synthesis pathway

To investigate the impact of *CcDFR* and *CcOMT9* on genes related to the cyanidin synthesis pathway in *C. cicadae*, we extracted RNA at different growth stages and assessed expression levels of cyanidin-related genes using RT-qPCR. On the second day of culture, compared to the WT, there was a varying degree of down-regulation in genes related to cyanidin synthesis of *ΔCcDFR* mutant (around 0.05, 0.46, 0.44, 0.01, 0.13, and 0.40-fold, respectively), indicating that deletion of *CcDFR* could reduce the expression of other genes in the cyanidin synthesis pathway and affect normal cyanidin synthesis. Compared with day 2, the expression levels of genes related to cyanidin synthesis were down-regulated to varying degrees in the WT cultured on day 6 (around 0.07, 0.56, 0.74, 1.49, 0.34, and 0.51-fold, respectively). This suggests that when cyanidin accumulation reaches a certain threshold, the genes involved in its synthesis may be affected by its content and undergo feedback inhibition. The genes associated with cyanidin synthesis are up-regulated to varying degrees in the *ΔCcOMT9* mutant on both day 2 and day 6 of culture compared to the WT (around 1.12, 1.54, 1.34, 1.06, 2.24, and 1.16-fold for day 2 and 2.26, 2.24, 1.08, 1.29, 1.12, and 1.15-fold for day 6, respectively) (Table 1). On day 6, the expression levels of genes related to cyanidin biosynthesis exhibited varying degrees of reduction in *ΔCcOMT9* mutant compared to day 2, except FUN_008622 (around 0.14, 0.81, 0.59, 1.78, 0.17, and 0.50-fold, respectively), potentially suggesting that excessive cyanidin production exerts a feedback inhibition effect on the expression of relevant synthetic genes.

**Table 1:** The effect of *CcDFR* and *CcOMT9* on the expression of genes related to C3G synthesis in *C. cicadae*.

Gene ID	FUN_005098 (CHS)	FUN_004156 (CHS)	FUN_001810 (CHI)	FUN_008622 (F3H)	FUN_000648 (LDOX)	FUN_007567 (bHLH)
WT (2d)	5.43 ± 0.29	3.04 ± 0.26	21.38 ± 1.61	21.99 ± 0.14	0.76 ± 0.12	1.89 ± 0.15
WT (6d)	0.38 ± 0.06	1.70 ± 0.04	15.77 ± 1.23	32.64 ± 0.15	0.26 ± 0.02	0.96 ± 0.12
*ΔCcDFR* (2d)	0.27 ± 0.03^**^	1.39 ± 0.11^**^	9.47 ± 0.36^*^	0.24 ± 0.02^**^	0.10 ± 0.01^**^	0.75 ± 0.21^*^
*ΔCcDFR* (6d)	1.12 ± 0.21^*^	2.29 ± 0.27^*^	11.45 ± 0.96^*^	2.46 ± 0.33^**^	0.08 ± 0.02^**^	0.66 ± 0.18^*^
*ΔCcOMT9* (2d)	6.08 ± 0.25*	4.69 ± 0.15*	28.68 ± 1.34*	23.46 ± 0.51*	1.70 ± 0.18**	2.19 ± 0.71*
*ΔCcOMT9* (6d)	0.86 ± 0.12*	3.81 ± 0.14*	17.01 ± 0.31*	41.99 ± 0.31*	0.29 ± 0.04	1.10 ± 0.42

### The metabolomics analysis of *ΔCcDFR* and *ΔCcOMT9* mutants revealed the variation in levels of C3G and phenylalanine

**Figure 6:**
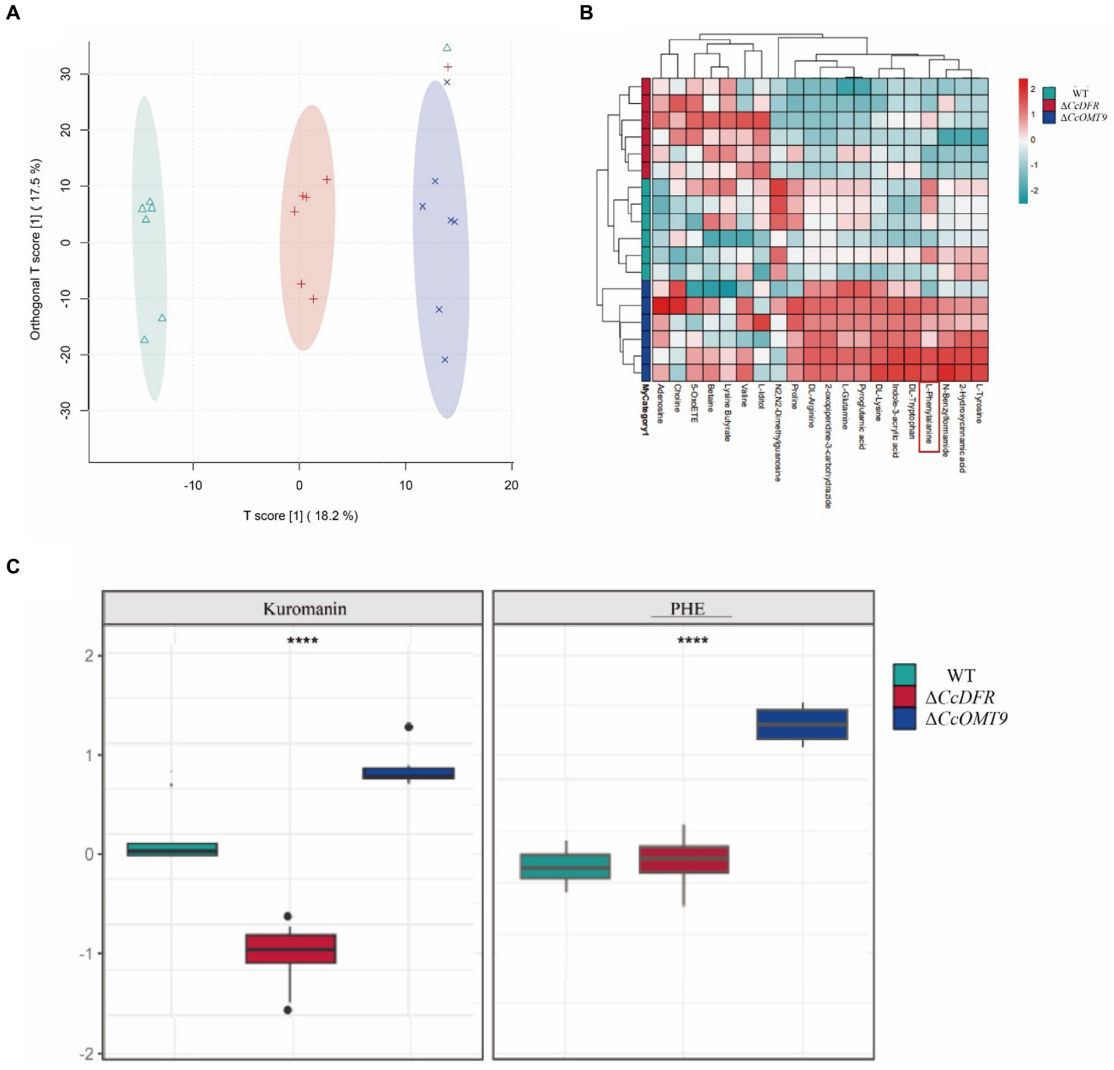
The analysis of differential metabolites of different mutants. **(A)** PCA analysis of total samples: scattered dots of different colors represent samples from different experimental groups; **(B)** Overall cluster plot of the samples in each group; **(C)** ANOVA boxplot.

The PCA results revealed a distinct separation trend among the WT, *ΔCcDFR*, and *ΔCcOMT9* mutants on the two-dimensional plot, with all strains exhibiting aggregation within this group (Figure 6A). The results indicate significant differences among the WT, *ΔCcDFR*, and *ΔCcOMT9* mutants, with high stability observed. The results of the cluster heat map analysis revealed distinct differences among the WT, *ΔCcDFR*, and *ΔCcOMT9* mutants, with a consistent and highly aggregated trend in metabolite accumulation observed within each group, and the phenylalanine content was significantly up-regulated in *CcOMT9* mutants (Figure 6B). After comparing the differential metabolites among the WT and *ΔCcDFR* and *ΔCcOMT9* mutants, significant differences were observed in flavonoids, including phenylalanine (a precursor to cyanidin synthesis) and cyanidin. The C3G (kuromanin) content of the *ΔCcDFR* mutant was found to be significantly lower than that of the WT and *ΔCcOMT9* mutant, while the phenylalanine content of the *ΔCcOMT9* mutant was significantly higher than that of the WT and *ΔCcDFR* mutant (Figure 6C).

### Combined metabolomics and transcriptome analysis suggested that *CcOMT9* affected the synthesis of cyanidin through modification of RNA

We conducted transcriptome sequencing to further compare the difference between WT and *ΔCcOMT9* mutants. Among them, 541 were up-regulated and 756 were down-regulated among a total of 1,297 differential genes between the WT and *ΔCcOMT9* mutant (Supplementary Figures S3A,B). KEGG analysis showed that 105 DEGs were enriched between the WT and *ΔCcOMT9* mutant, and the metabolism pathway mainly involved in the cell cycle, flavonoid biosynthesis, etc. was analyzed. The top 20 metabolic pathways with significant enrichment (*p* < 0.05) were selected for display in an enrichment bubble map, and four of these pathways were found to be related to anthocyanin biosynthesis (Supplementary Figure S3C). The GO (Gene Ontology) enrichment analysis was carried out on the DEGs of the two treatment groups (Supplementary Figure S3D). Among biological processes, DEGs are primarily concentrated in cellular processes, metabolic processes, biological regulation, regulation of biological processes, response to stimulus, and so on. In terms of cell components, DEGs are mainly found in the cellular anatomical entity, protein-containing complex, ribonucleoprotein component, and so on. In molecular function, DEGs are mainly enriched in binding, catalytic activity, transporter activity, transcription regulator activity, and ATP-dependent activity. The down-regulation of 9 out of 10 genes involved in the phenylalanine metabolism pathway suggests a blockage in the *ΔCcOMT9* mutant. This blockage leads to the accumulation of phenylalanine and an increase in cyanidin biosynthesis ability. This conclusion is supported by the metabolomics analysis, which shows the accumulation of phenylalanine in the *ΔCcOMT9* mutant.

To validate the precision of RNA-seq sequencing analysis outcomes, we picked 15 genes from the DEGs across the four metabolic pathways (Supplementary Table S3) and tested the expression of these genes after different treatments by RT-qPCR. The results of RT-qPCR detection were basically consistent with those of RNA-seq, which confirmed the reliability of transcriptome data (Supplementary Figure S4).

The differences in mRNA methylation between WT and *ΔCcOMT9* mutants were analyzed to check if *CcOMT9* can be responsible for mRNA methylation in *C. cicadae*. Analysis of the relative methylation rate showed there were 401 up-regulated and 243 down-regulated differential peaks between WT and *ΔCcOMT9* mutant (Supplementary Figures S5A,B). Functional enrichment analysis of genes related to differential peaks was conducted using GO and KEGG analysis. GO analysis clustered into the cellular process and metabolic process in biological process, cellular anatomical entity and protein-containing complex in cellular component and binding and catalytic in molecular function activity (Supplementary Figure S5D). KEGG pathway enrichment analysis clustered to phosphatidylinositol signaling system, MAPK signaling pathway-yeast, and aminoacyl-tRNA biosynthesis (Supplementary Figure S5C).

## Discussion

*Cordyceps cicadae* is a significant biological resource that serves as a prominent ingredient in food and medicine, with anti-tumor and other health benefits. Additionally, it can inhibit bacteria growth and act as an insecticidal fungus for plant protection. Previous research showed that *C. cicadae* strains can produce specific proteins (Cen et al., 2021) and polysaccharides (Zhang et al., 2017) with antibacterial properties. In this study, we discovered that *C. cicadae* can produce C3G, a common anthocyanin in plants that possesses inhibitory properties against a diverse range of pathogenic bacteria, making it an invaluable biological resource.

**Figure 7:**
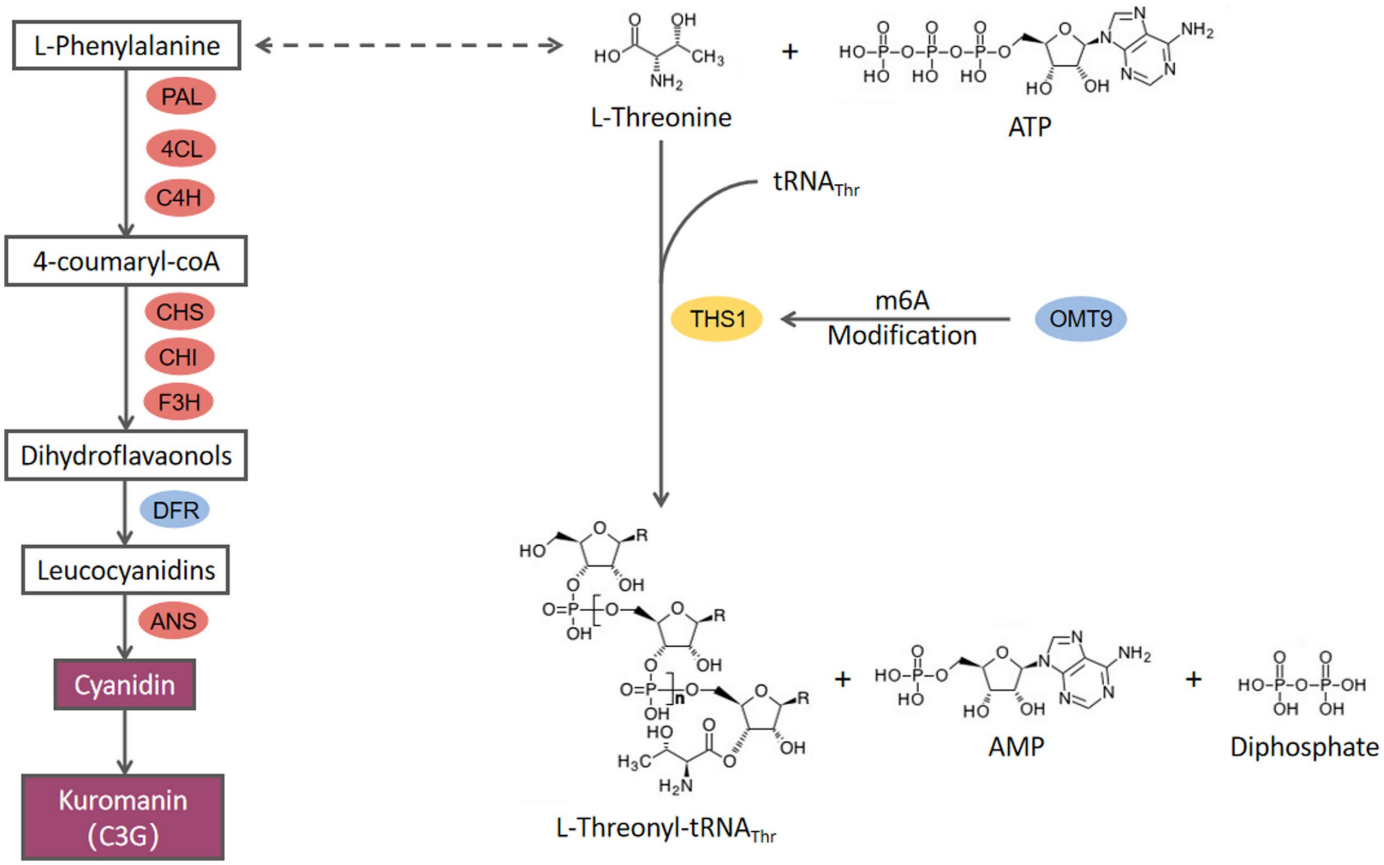
The proposed model for the molecular mechanism of *CcDFR* and *CcOMT9*.

Cyanidin is the most widely distributed and important source of anthocyanins that exhibits anti-tumor and antibacterial properties although the focus has been on plants rather than fungi. Bu et al. first reported that five anthocyanins, including cyanidin 3-O-glucoside, can be produced by the fungus *Aspergillus sydowii* H-1. The anthocyanin synthesis pathway was also investigated in detail (Bu et al., 2020). They demonstrated that this is a novel anthocyanin synthesis pathway in fungi, which takes different forms in plants, and three lncRNAs (long non-coding RNAs) are involved in the anthocyanin biosynthesis pathway. In the current research, we discovered that *C. cicadae* can produce cyanidin, but we did not find any lncRNAs regulatory genes in *C. cicadae* to regulate anthocyanin biosynthesis ability. We also did not find UGT homolog in *C. cicadae* which catalyzes cyanidin to biosynthesize other anthocyanins besides cyanidin 3-O-glucoside. These results indicate that there is a unique anthocyanin biosynthesis pathway in *C. cicadae* (Figure 7). This hypothesis is confirmed by metabolomics results that no oenin or malvidin-3G were found.

*CcDFR* is the key gene in cyanidin 3-O-glucoside biosynthesis in *C. cicadae*. In plants, dihydroflavonol-4-reductase (DFR) is the first key enzyme in anthocyanin synthesis, and its content in plants can determine the colorization of flowers and fruits both directly and indirectly (Lim et al., 2016; Wang et al., 2017a). In this study, *CcDFR* affected the synthesis of cyanidin in *C. cicadae*. *CcDFR* deletion does not only lead to cyanidin-3-O-glucoside biosynthesis blocking but also causes the expression of the upstream genes in the anthocyanin biosynthesis pathway to dramatically down-regulate, indicating that the expression of *CcDFR* could negatively regulate the activity of cyanidin biosynthesis pathway. *CcDFR* exhibits diverse biofunctions in *C. cicadae*, such as being involved in conidia growth and synnemata formation. Meanwhile, the increasing sensitivity of the *ΔCcDFR* mutants to cell-interfering agents suggests that the deletion of this gene may impair the ability to withstand adverse environmental factors. These results extend our understanding of the mechanisms of fungal development.

The function of *CcOMT9* may affect phenylalanine content, thereby influencing cyanidin biosynthesis although the growth rate and conidial number of *ΔCcOMT9* mutant did not differ with the wild type. Meanwhile, we observed a decrease in the adaptability of *C. cicadae* to the environment, along with an increase in chitinase activity in the *ΔCcOMT9* mutant. These findings suggest that while the *CcOMT9* gene may enhance natural adaptation to adverse conditions, it may also weaken the insect parasitism ability, highlighting a delicate balance between adaptation and parasitism. The antibacterial ability of *ΔCcOMT9* mutant was improved, offering a novel perspective for eco-friendly biological control of pathogenic bacteria and facilitating further utilization and exploration of *C. cicadae* resources.

Previous studies have demonstrated the prevalence of 3-methylcytidine (m3C) modifications in eukaryotic tRNAs. In yeast, 3-methylcytidine (m3C) occurred at the 32nd position, Thr, or Ser of the tRNA. Trm140 in *S. cerevisiae* can recognize the unique V-loop of tRNA_Ser_, or it may cooperate with other proteins (such as seryl-tRNA synthetase, Ses1) to identify the V-loop (Han et al., 2017). In mammal cells, the Trm140 family possesses two or three homolog members known as the METTL (methyltransferase-like) family; these proteins are involved in the methylation of particular tRNAs and mRNAs (Xu et al., 2017). In the present research, *CcOMT9* in *C. cicadae* is homologous to *Trm140* in *S. cerevisiae*, and mRNA methylation levels of threonyl tRNA synthetase (FUN_002718, *ths1*) are down-regulated in m6A data. We hypothesize that CcOMT9 and THS1 may jointly recognize the V-loop of tRNA_Thr_. The lack of *CcOMT9* in *C. cicadae* results in reduced m6A modification of *ths1* mRNA, leading to an accumulation of L-threonine which indirectly promotes the synthesis of phenylalanine and consequently increases cyanidin content. However, additional research is needed to elucidate.

Altogether, we found a new cyanidin biosynthesis pathway in the valuable fungus *C. cicadae*. Two important genes involved in cyanidin biosynthesis are investigated. Gene *CcDFR* is the key regulation gene in the cyanidin biosynthesis pathway, and gene *CcOMT9* regulates the cyanidin biosynthesis pathway by affecting phenylalanine metabolism. These two genes also have functions related to environmental adaptability and the regulation of growth and development. We found that *CcOMT9* is implicated in mRNA methylation, but the mechanism needs further exploration in future studies. Additionally, artificial fermentation could be utilized for the industrial production of cyanidin to overcome seasonal limitations and land resource shortages associated with this plant-based production.

## Conclusion

In this study, the mechanisms underlying the biosynthesis of cyanidin-3-O-glucoside (C3G) by *CcDFR* and *CcOMT9* were investigated. We also explored the effects of deleting these two genes on hyphal growth, sporulation, fruiting body formation, the content of the active ingredient, and mycelial growth under chemical stress. The results proved that the deletion of the *CcDFR* gene directly inhibits cyanidin synthesis by reducing DFR enzyme content. Meanwhile, *CcOMT9* indirectly affects cyanidin synthesis by regulating phenylalanine (precursors to anthocyanin synthesis) metabolic pathways by tRNA modification.

## Data availability statement

The datasets presented in this study can be found in online repositories. The names of the repository/repositories and accession number(s) can be found in the article/Supplementary material.

## Author contributions

ZZ: Writing – review & editing, Writing – original draft, Methodology, Data curation, Conceptualization. YZ: Writing – original draft, Methodology, Investigation, Data curation. WC: Writing – review & editing, Supervision, Resources, Funding acquisition. F-CL: Writing – review & editing, Supervision. HW: Writing – review & editing, Supervision, Funding acquisition, Conceptualization.
