# Supplementary material for: Roles of CcDFR and CcOMT9 in the cyanidin biosynthesis and development of Cordyceps cicadae
Source: Front Microbiol. 2024 Mar 6;15:1353710. doi: 10.3389/fmicb.2024.1353710 (PMC10953825; doi:10.3389/fmicb.2024.1353710)
Supplement: Supplementary file 1 [file Data_Sheet_1.docx]

Supplementary materials 1: Supplementary figures


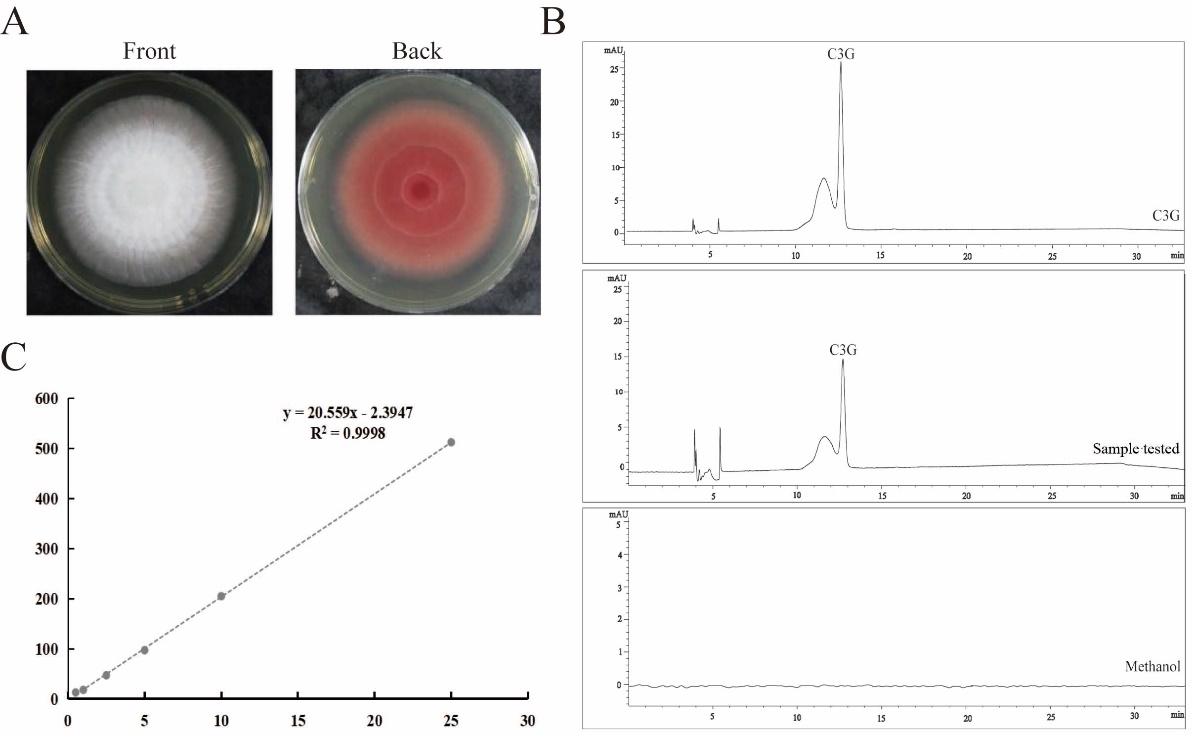


Fig. S1 The establishment of detection system of C3G in *C. cicadae*.

A. The colony morphology of *C. cicadae* strain 2-2 on PDA. B. The plot of C3G HPLC: HPLC of standard, sample tested, and methanol. C. The standard curve of C3G.


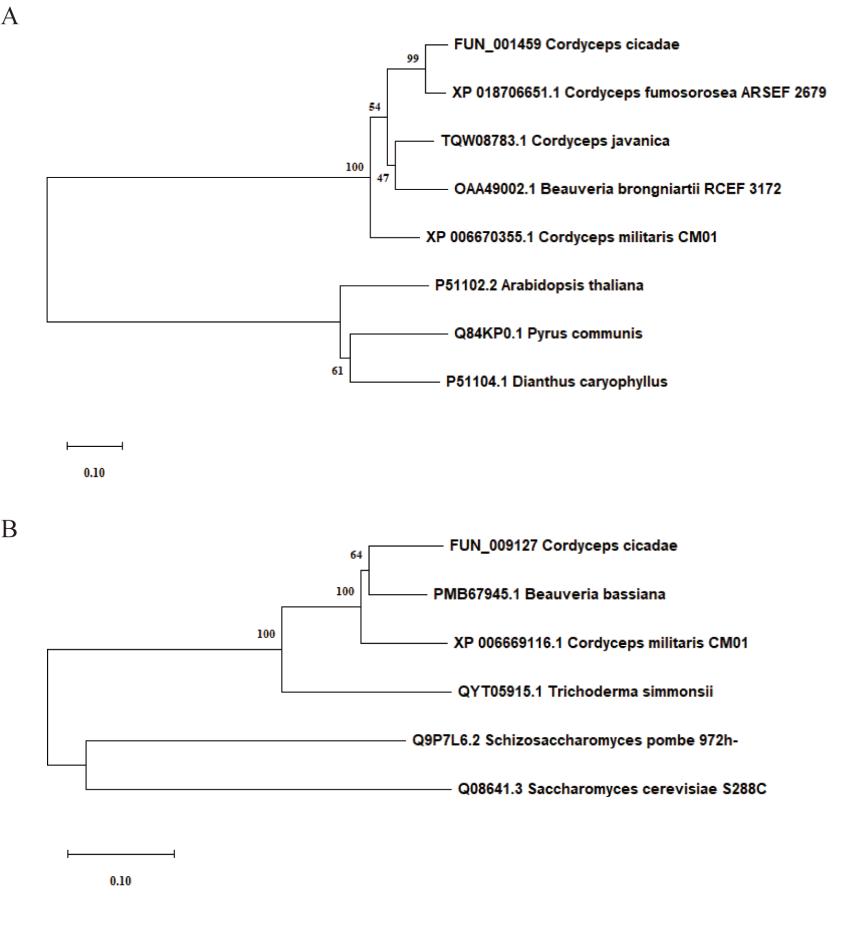


Fig. S2 The phylogenetic trees constructed by amino acid sequences.

A. Phylogenetic tree of CcDFR (FUN_1459). B. Phylogenetic tree of CcOMT9 (FUN_9127).


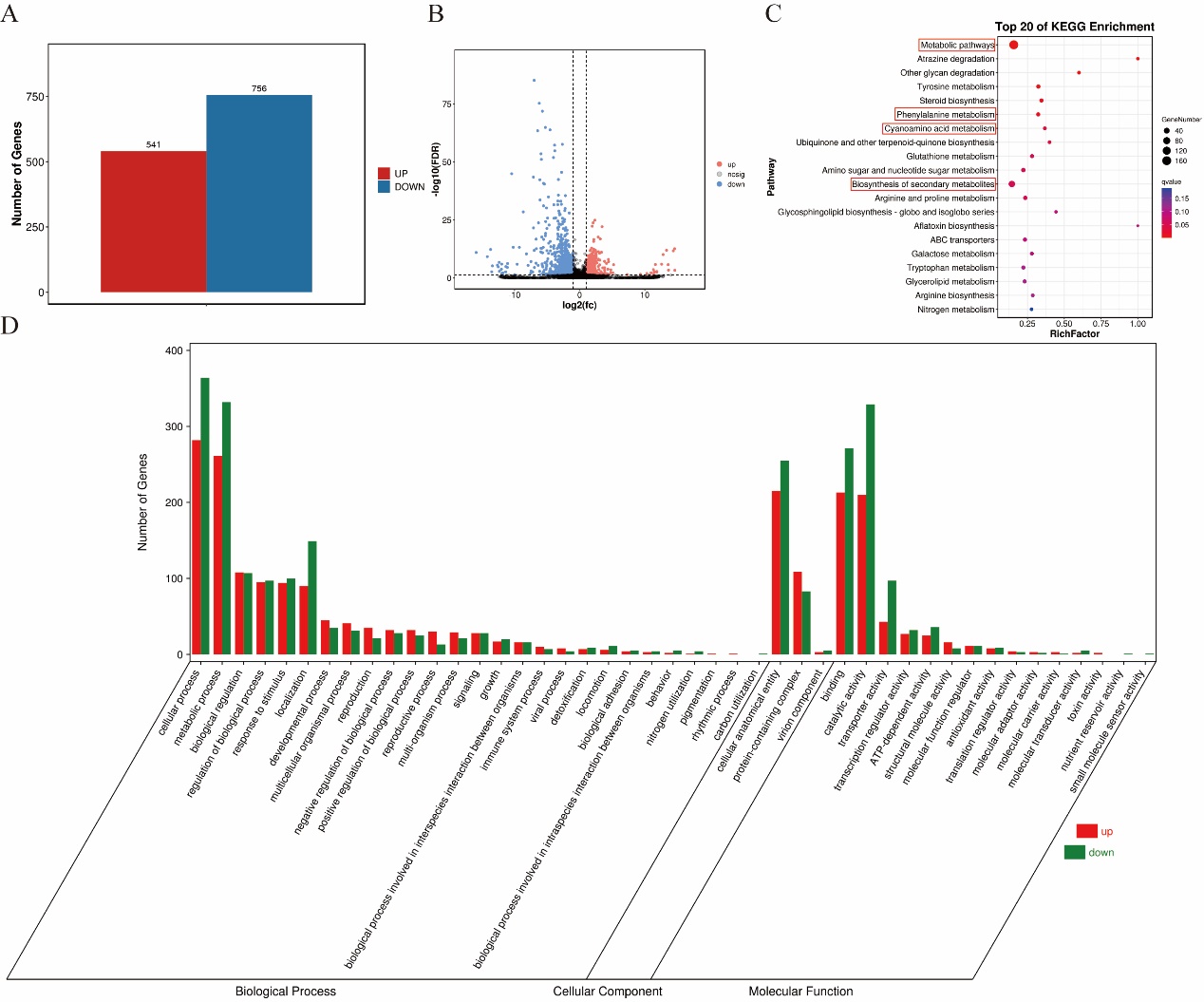


Fig. S3 The analysis of differentially expressed genes between WT and *ΔCcOMT9* mutant.

A. The statistical map of DEGs between WT and *ΔCcOMT9* mutant; B. The volcano plot of DEGs between WT and *ΔCcOMT9* mutant: The red dots indicate up-regulation and the blue dots indicate down-regulation, while the gray ones indicate no significant difference; C. KEGG pathway enrichment analysis: The first 20 pathway with the smallest Q value are used, the ordinate is pathway and the abscissa is the enrichment factor (RichFactor equals the number of differential genes in this pathway is divided by all numbers in this pathway). Size indicates the number and the redder the color is, the lower the Q value is; D. GO function analysis of DEGs between WT and *ΔCcOMT9* mutant: The x-axis represents the second-order GO term, while the y-axis indicates the number of DEGs associated with this term. The red dots indicate up-regulation, while the green ones indicate down-regulation.


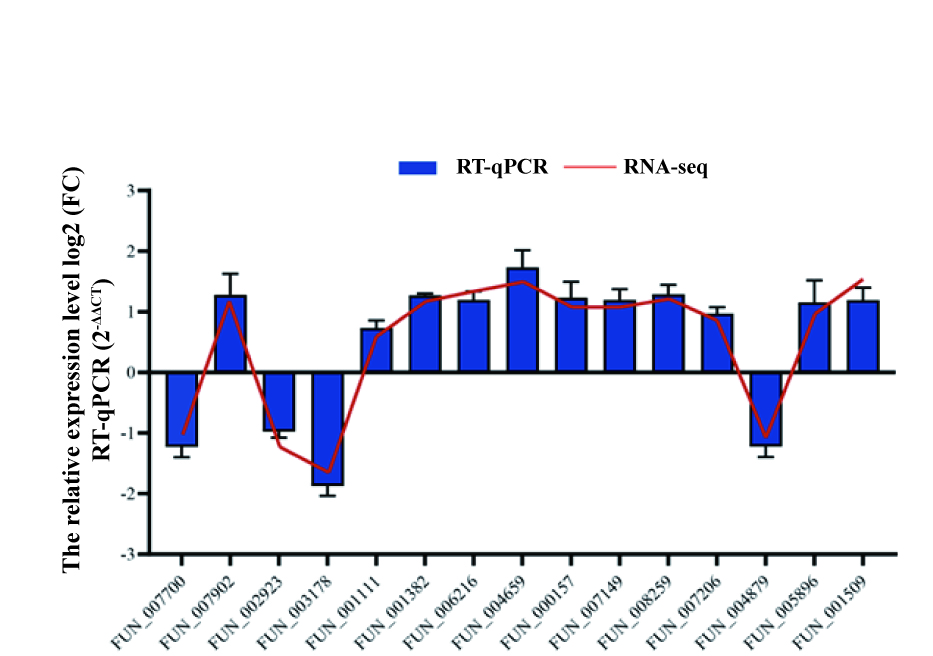


Fig. S4 The expression level of 15 genes in RNA-sequencing and RT-qPCR detection. (

The horizontal axis denotes the 15 genes, while the vertical axis represents the logarithmic value of fold change (log2(FC). The blue histogram represents results from RT-qPCR and error bars indicate the standard deviation. The red line represents results from RNA-Seq.


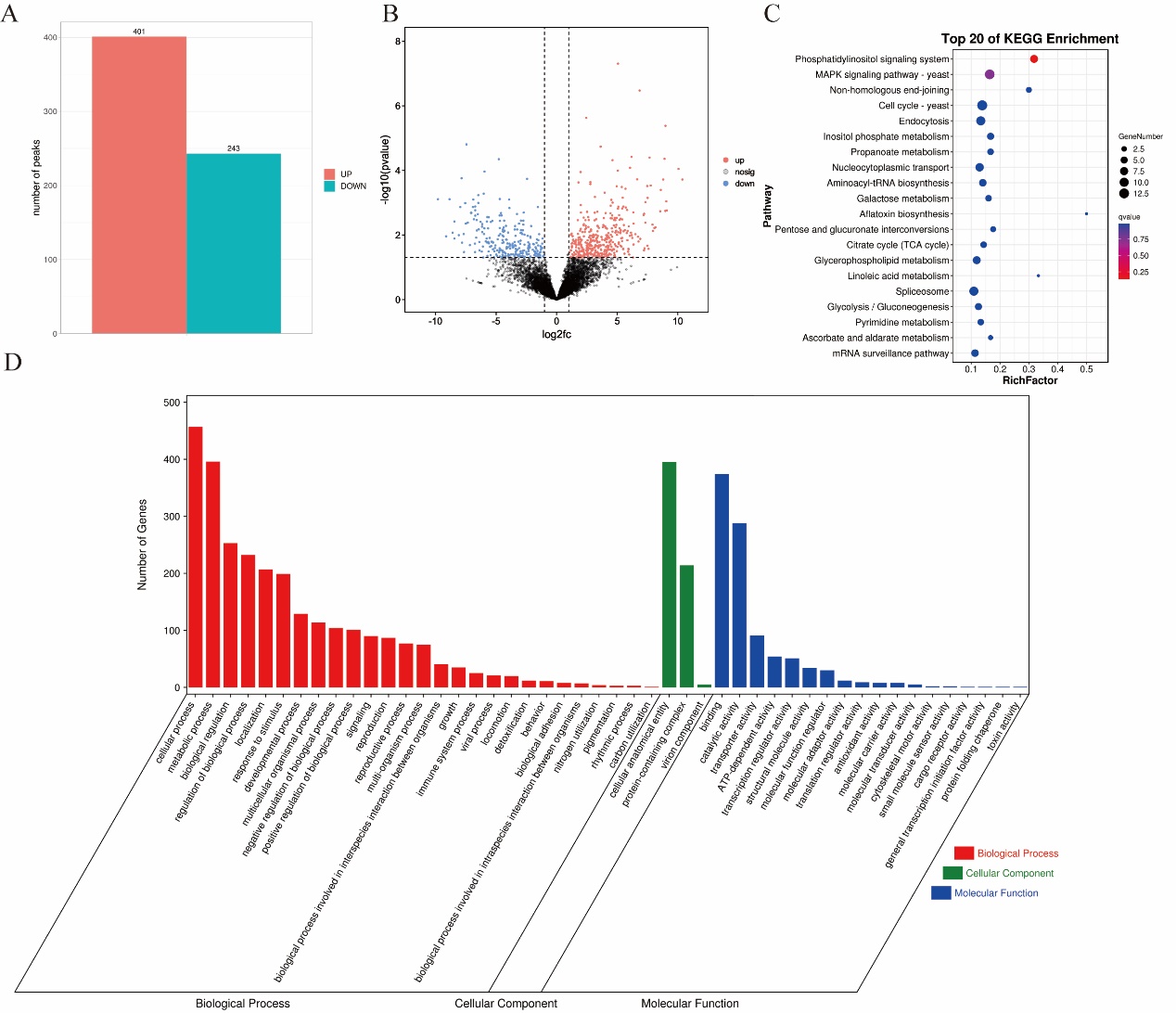


Fig. S5 The differential analysis of methylation rate at peak related genes between the WT and *ΔCcOMT9* mutant.

A. The statistical map of differential peaks between WT and *ΔCcOMT9* mutant; B. The volcano plot of peak related genes between WT and *ΔCcOMT9* mutant (P < 0.05 and |log2FC| > 1C); KEGG pathway enrichment analysis; D. GO function analysis of differential peaks between WT and *ΔCcOMT9* mutant
